# Global research trends in prediction of difficult airways: A bibliometric and visualization study

**DOI:** 10.1097/MD.0000000000033776

**Published:** 2023-05-12

**Authors:** Xiaoyan Li, Yixiao Lian, Fang Pan, Hong Zhao

**Affiliations:** a Department of Anesthesiology, Peking University People’s Hospital, Beijing, China; b Department of Library, Peking University People’s Hospital, Beijing, China.

**Keywords:** assessment and prediction of difficult airway, bibliometrics, global trends, visualization research

## Abstract

Many tools are used to predict difficult airway, including bedside screening tests, radiological variables, and ultrasonography. However, the “gold standard” to identify difficult airway before intubation has not been established. The assessment and prediction of difficult airway is receiving increasing attention in clinical practice due to the devastating results of failed oxygenation or intubation. A literature visualization study is necessary to understand the research trend and help tailor future research directions. Science citation index-expanded web of Science database were used to search for literature related to assessment and prediction of difficult airways published before May 9th, 2022. VOS viewer software was used for visual analysis, including literature statistics, and co-occurrence analysis. A total of 2609 articles were included. The amount of relevant research interest and literature is increasing every year. According to co-occurrence network analysis, the research results can be grouped into the following 5 clusters, intubation approaches, intubation in special populations, difficult airway assessment tests, intubation in critical care/emergency settings and education, and laryngoscopes. Co-occurrence overlay analysis showed that video laryngoscopes and index prediction (including computed tomography and ultrasonography), emerged recently and comprised an important percentage of current studies. It can be predicted that future studies should focus on understanding the upper airway anatomy and constructing risk index predictions. Based on current global research trends, risk index predictions are the next hot topics in the evaluation and prediction of difficult airways, and video laryngoscopes will continue to be a hot topic in this field.

## 1. Introduction

The American Society of Anesthesiologists defines a difficult airway as “a conventionally trained anesthesiologist experiences difficulty with facemask ventilation of the upper airway, difficulty with tracheal intubation, or both”.^[1]^ Difficult airways occur due to a number of factors, including airway anatomical and physiological variation, such as broken neck, and buckteeth,^[2]^ cervical spine ankylosis,^[3]^ and temporomandibular joint ankylosis et al^[4]^.

According to American Society of Anesthesiologists practice guidelines, before the initiation of anesthetic care or airway management, an airway physical examination should be conducted in order to identify physical characteristics of a potential difficult airway. The physical examination may include assessment of facial features and assessment of anatomical measurements and landmarks.^[5]^ Radiological parameters such as the distance between C1 and C2 spinous processes were suggested as indicators of difficult airways.^[6]^ Ultrasonic measuring of arm-chin distance, mandible length, and thickness of soft tissue from skin to thyroid were also found to be associated with difficult laryngoscopy.^[7]^ However the “gold standard” to identify difficult airways before intubation has not been established. Therefore, it is necessary to summarize the current status of research on the assessment and prediction of difficult airways, and predict promising keywords and trends.

Visual analysis combines new theory-based tools with innovative interactive techniques and visual representations to enable an information discourse. Visualization transforms data into a readable form, highlighting important features of the data, including commonalities and anomalies. These visual representations allow the readers to quickly perceive outstanding aspects of the data. Visual representations enhance the cognitive reasoning process, making the analysis process faster and more focused. This approach has been successfully used to assess research trends in spine,^[8]^ sepsis,^[9]^ diabetes,^[10]^ osteoarthritis,^[11]^ and shoulder-related pain syndrome.^[12]^ However, to our Acknowledgments, the quantity and quality of studies evaluating the prediction of difficult airways have not been visualized. Therefore, the purpose of this study is to evaluate the current status and trends of research in difficult airway prediction.

## 2. Materials and methods

### 2.1. Data source

Bibliometric analysis was based on science citation index-expanded of Web of Science (WoS), which covered the vast majority of biomedical literature. Therefore, it is comprehensive and representative.

### 2.2. Retrieval strategy

All literature was retrieved in WoS from database creation until May 9th, 2022. The test formula of this research is as follows: Topic = [(“difficult airway” OR “difficult facemask ventilation” OR “difficult laryngoscopy” OR “difficult intubation” OR “failed intubation”) AND (assessment OR evaluat* OR predict*)] OR (“airway management” AND “difficult airway”) AND language = (English).

### 2.3. Data collection

All records of each publication, including title, author’s names, abstract, keywords, name of publishing journal, nationalities, affiliations, were downloaded as Text files from the WoS database.

### 2.4. Visual analysis

VOS viewer, a freely available computer program for bibliometric mapping, was used for visual analysis, including literature statistics,co-writing, co-occurrence, citation, document coupling, and co-citation of the literature in this study.^[13]^ The maps of co-occurrence network visualization, co-occurrence overlay visualization, and co-occurrence density of visualization were created based on textual data. Global publishing trends show the number and trends of global publications and contribution of countries, journals, research areas, and authors.

Co-occurrence analysis is a method to build the relationship of keywords from the publications. Strongly correlated keywords were located close to each other and could be grouped into different clusters through co-occurrence network visualization. The co-occurrence overlay visualization presents the keywords according to when the words were published, which is of great significance to visualize the trend in the difficult airway research over time. In co-occurrence density visualization, the higher weights of the neighboring items, the closer the color of the point is to orange.

Ethical approval was not required because only published literature data were collected in this study.

## 3. Results

### 3.1. Global publishing trends

#### 3.1.1. Number and trends of global publications

**Figure 1.**
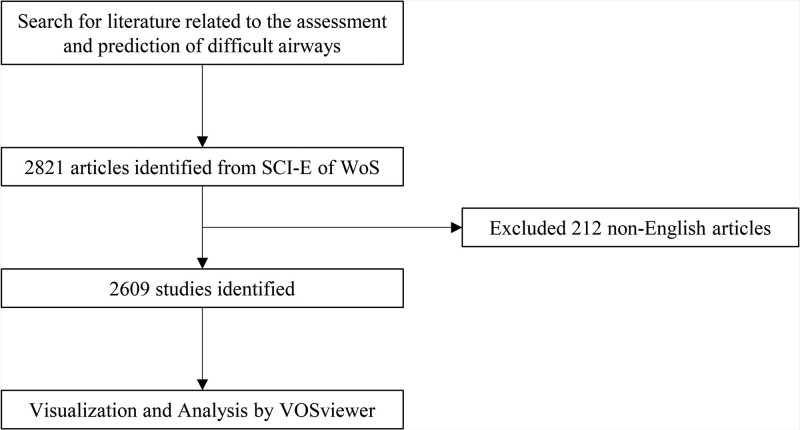
Publications screening flowchart.

**Figure 2.**
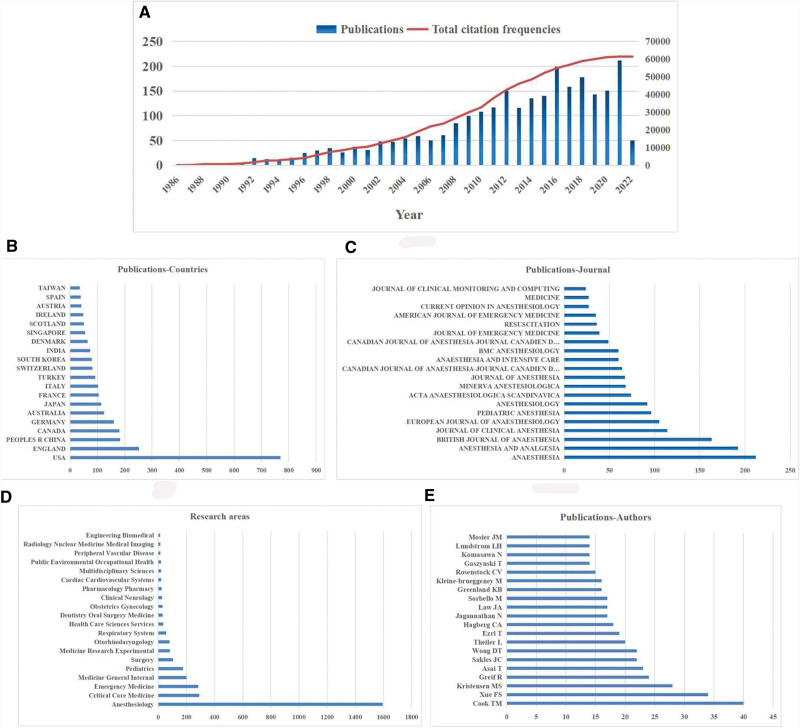
Global publishing trends. (A) Number and trends of global publications, showing the increase of total citations frequency over the years. (B) Contribution of countries assessed by most cited publications. (C) Journals with most publications. (D) Most related research areas. (E) Top 20 authors in difficult airway field.

We retrieved 2821 articles published from 1986 to May 9th, 2022. When 212 non-English were excluded, a total of 2609 articles met the search criteria (Fig. 1). In terms of the annual number of articles published, the number of articles published in 2021 was the highest, which was 211. Since 1986, there has been a gradual increase in the number of global publications. The frequency of total citations was also increased over the years (Fig. 2A).

#### 3.1.2. Contribution of countries

In terms of the assessment and prediction of difficult airway, the United States had the largest number of articles and consisted largest percentage of publications (770, 28.99%), followed by England (252, 9.49%), China (183,6.89%), Canada (181, 6.81%), and Germany (229, 6.02%) (Fig. 2B).

The United States has the most total times cited (17,202), followed by the United Kingdom (10,350), France (5119), Canada (4877) and Germany (3819).

### 3.2. Analysis of global publications

#### 3.2.1. The journals

The anaesthesia [impact factor (IF) = 6.955, 2020] published the largest number of studies (212). There were 192 articles in anesthesia and analgesia (IF = 5.178, 2020), 163 articles in *British Journal of Anaesthesia* (IF = 9.166, 2020), 114 articles in *Journal of Clinical Anesthesia* (IF = 9.452, 2020), and 105 articles in *European Journal of Anaesthesiology* (IF = 4.33, 2020) on difficult airway. The top 20 journals that published the largest number of articles are listed in Figure 2C.

#### 3.2.2. Research areas

Anesthesiology was the most popular research field (1595,61.13%), followed by critical care medicine (290,11.11%), emergency medicine (284,10.89%), medicine general internal (201,7.70%), pediatrics (176,6.75%) (Fig. 2D).

#### 3.2.3. Authors

Cook TM was identified as the most productive author with 40 papers, and Xue FS (34 papers) ranked second, followed by Kristensen MS (28 papers), Greif R (24 papers), and Asai T (23 papers). The top 20 authors in the difficult airway field were shown in Figure 2E.

### 3.3. Co-occurrence analysis

Co-occurrence analysis is a method to build the relationship of keywords from the publications. The trends and current topics of the research areas were established by the keywords network map, which was created by the VOS viewer (the minimum number of occurrences of a keyword was over 5 among all the publications). Strongly correlated keywords were located close to each other, and less strongly correlated terms were located far away from each other.

#### 3.3.1. Co-occurrence network visualization

**Figure 3.**
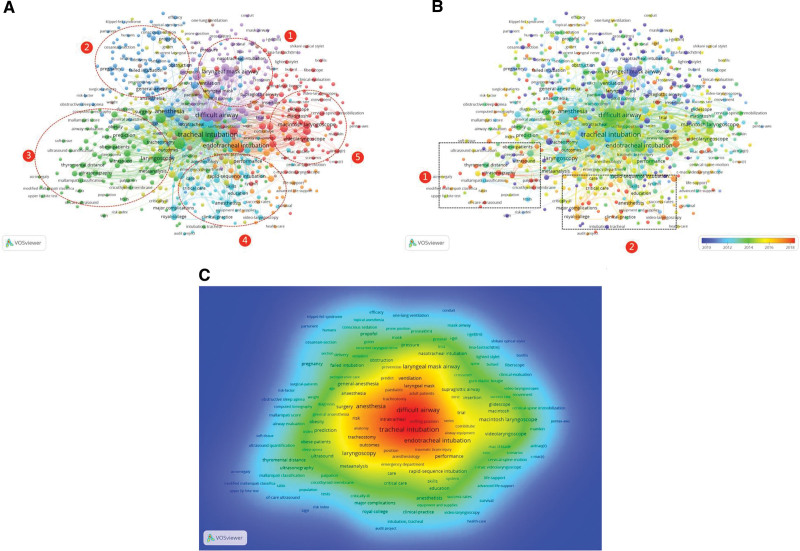
Co-occurrence analysis. (A) Co-occurence network visualization. Network visualization analysis arranges keywords that are closely related with each other in 1 area (a circle), and gives these keywords 1 single color. Keywords were classified into 5 clusters shown in different colors, i.e., cluster 1, intubation approaches, cluster 2, intubation in special population, cluster 3, difficult airway assessment tests, cluster 4, intubation in critical care/emergency settings, and cluster 5, laryngoscopes. (B) Co-occurence of overlay visualization. The overlay visualization presents the keywords according to when the words were published. The color blue represented the words that appeared relatively earlier, while the keywords in orange appeared more recently. Items in box 1 (computed tomography, ultrasonography, and index prediction) and box 2 (video laryngoscope) represented mos recently emerged items. (C) Co-occurence density visualization. Color of each point depends on the density of items at that point. The larger the number of items in the neighborhood of a point and the higher the weights of the neighboring items, the closer the color of the point was to orange.

There were altogether 4871 items automatically retrieved from 2609 articles, with 624 items qualified as keywords because they appeared more than 5 times among all the publications. Each keyword is 1 dot, and the size of dot represents publishing frequency, that is, the larger the dot, the more frequent this keyword appeared in all these articles. Network visualization analysis arranges keywords that are closely related with each other in 1 area (a circle), and gives these keywords 1 single color, such as red (Fig. 3A). For example, color red represents keywords related with 1 theme, that is, laryngoscopes. The largest red dot was video laryngoscope, which means the most popular research topic in laryngoscope cluster was video laryngoscope.

After removal of “endotracheal intubation,” “difficult airway,” and “airway management,” keywords can be classified into 5 clusters shown in different colors, that is, cluster 1, intubation approaches (Laryngeal mask airway [for ventilating a patient] and nasal tracheal intubation), cluster 2, intubation in special population (including patients undergoing general anesthesia for cesarean section), cluster 3, difficult airway assessment tests (including ultrasonography, Mallampati classification and risk factor index), cluster 4, intubation in critical care/emergency settings and education cluster, and cluster 5, laryngoscopes.

In the intubation approaches cluster, the main keywords were laryngeal mask airway, i-gel, nasal tracheal intubation and supraglottic airway.

In the intubation in special population, the main keywords were pregnancy and cesarean section.

In difficult airway assessment tests cluster, the main keywords were index prediction, airway evaluation, Mallampati score, upper lip bite test, thyromental distance and ultrasound quantification.

In intubation in critical care/emergency settings and education cluster, the main keywords were critical care, emergency patient and education.

In the laryngoscopes cluster, the main keywords were video laryngoscope, airtraq, glidescope, and fiberscope.

#### 3.3.2. Co-occurrence overlay visualization

The overlay visualization is identical to the network visualization except that items are colored differently. The overlay visualization presents the keywords according to when the words were published, with different colors indicating different years of publication. The color blue represented the words that appeared relatively earlier, while the keywords in orange appeared more recently. This method is of great significance to visualize the trend in the difficult airway research over time (Fig. 3B).

Color orange to red indicated the most recent literature and the color blue indicates studies published before the year 2014 in our study. Keywords in cluster 1, intubation approaches, and cluster 2, intubation in special population (clusters identified through network visualization) were main topics before 2014. Publications in difficult airway assessment tests (cluster 3) represented the most recent literature as the dots being orange in the co-occurrence overlay analysis, because there is no confirmative answer for prediction of a difficult airway. Computed tomography, ultrasonography, and index prediction emerged recently and size of the dots were all relatively large shown in box 1 (Fig. 3B). Studies focused on video laryngoscope emerged from the year 2010 and remain a research hot topic for many years shown in box 2 (Fig. 3B). It can be predicted future studies should focus on understanding the upper airway anatomy and risk index predictions.

#### 3.2.3. Co-occurrence density visualization

In the item density visualization, items are indicated by their label in a similar way as in the network visualization and the overlay visualization. In the VOS viewer, each point in a map has a color that depends on the density of items at that point. The larger the number of items in the neighborhood of a point and the higher the weights of the neighboring items, the closer the color of the point is to orange. The orange area contained these keywords: difficult airway, tracheal intubation, anatomy, airway equipment, predict, prevention, risk, and outcomes.

Co-occurrence density analysis in this study revealed that index prediction and video laryngoscope are both between color green and color yellow (Fig. 3C), which means these 2 items are important keywords and being investigated frequently.

## 4. Discussion

This visualization study about difficult airway literature revealed that the annual number of publications increased dramatically in recent 20 years. As no single assessment test can perfectly predict difficult airways, the risk index predictions of difficult airway will be a hot topic, and video laryngoscope remains a hot topic too.

A country’s total number of citations represents its academic influence and the quality of its publications. The United States has the highest contribution and influence in terms of the total number of papers published and citation profiles. So the United States is the leader in this field. China ranks the fifth in literature coupling link strength, but it does not rank among the top 10 countries in total citations. This implicates that China only contributes to the quantity in the field of academic research, while the quality needs to be improved.

Difficult airway assessment tests (such as Mallampati classification^[14]^), computer tomography,^[15,16]^ ultrasonography,^[17,18]^ and risk index predictions^[19–21]^ were identified as future research trends and hot topics through co-occurrence analysis. These topics were studied frequently (big dot size in co-occurrence network visualization), emerged in recent years (orange color in co-occurrence overlay analysis), and with relative importance (color green and yellow in density analysis). Therefore, it can be predicted that airway anatomy examination and risk index predictions are promising prediction tools for difficult airway and require further studies.

Risk index predictions should be investigated because multiple airway features should be obtained to determine a patient’s potential for a difficult airway. Firstly, bedside screening test are not well suited for the purpose of detecting unanticipated difficult airways. A systematic review involving 844,206 patients from 133 studies investigated the sensitivity and specificity of bedside airway screen tests, and the average sensitivity ranged from 24% (thyromental distance) to 51% (modified Mallampati test) and average specificity ranged from 87% (modified Mallampati test) to 93% (mouth opening test).^[5]^ Secondly, a combination of several risk factors are indicated. According to the Multicenter Perioperative Outcomes Group, involving 176,679 cases, there were 11 dependent predictors of difficult mask ventilation combined with difficult laryngoscopy, including age 46 years or more, body mass index 30 or more, male sex, Mallampati III or IV, et al^[22]^ Thirdly, with the development of technology, parameters indentified through airway ultrasonography and computed tomography convey some new information about airway anatomy, such as ultrasonic assessment of mandible length, and thickness of soft tissue from skin to thyroid.^[7]^ There are also radiological variables to identify the distance between C1 and C2 spinous processes and up to 21 parameters were compared between easy and difficult intubation patients.^[6]^

Video laryngoscope remains a hot topic since it appeared in around 2010 according to co-occurrence overly visualization because it is a group of useful tools to fulfill endotracheal intubation. A meta-analysis of 77 randomized controlled trials comparing video-assisted laryngoscopy with direct laryngoscopy in patients with predicted difficult airways reported improved laryngeal views, a higher frequency of successful intubations, a higher frequency of first attempt intubations, and fewer intubation maneuvers with video-assisted laryngoscopy. In addition, in the scenario of failed intubation using direct laygoscopy, it is suggested using the Airtraq, Bonfils, CTrach, GlideScope, McGrath, and Pentax AWS to achieve successful intubation.^[23]^ In recent years, video laryngoscopy has not only been used in adults,^[24]^ but also further explored in children and newborns.^[25]^

### 4.1. Advantages and limitations

Although this study evaluated the current status and trends of the research on the evaluation and prediction of difficult airways by means of visual analysis, the following limitations should be pointed out. Both WoS and Pubmed databases are English literature. Non-English literature was not included in the research scope, which may lead to language bias. In addition, there may be a discrepancy between reality and current results. For example, some recently published papers of high quality, with short publication time and low citation frequency, may not be highlighted. Therefore, in daily research work, we still need to pay attention to the latest original research literature and other non-English literature.

## 5. Conclusion

Based on current global research trends, risk index predictions are the next hot topics in the evaluation and prediction of difficult airways, and video laryngoscopes will continue to be a hot topic in this field.

## Acknowledgements

We thank Dr Dan Xing, M.D. from Arthritis Clinic & Research Center, Peking University People’s Hospital, Peking University, Beijing, China for his help with conduct of the study.

## Author contributions

**Data curation:** Xiaoyan Li, Yixiao Lian.

**Formal analysis:** Xiaoyan Li, Yixiao Lian, Hong Zhao.

**Funding acquisition:** Hong Zhao.

**Methodology:** Fang Pan, Hong Zhao.

**Project administration:** Fang Pan, Hong Zhao.

**Writing – original draft:** Xiaoyan Li, Hong Zhao.

**Writing – review & editing:** Hong Zhao.
